# Integrative analysis of tumor stemness and immune microenvironment deciphers novel molecular subtypes in hepatocellular carcinoma

**DOI:** 10.1016/j.gendis.2023.101077

**Published:** 2023-09-07

**Authors:** Zhiyi Wang, Shuang Chen, Dongxiao Li, Hui Xu, Siyuan Weng, Yuyuan Zhang, Yuqing Ren, Chunguang Guo, Xiuling Li, Zaoqu Liu, Xinwei Han

**Affiliations:** a Department of Interventional Radiology, The First Affiliated Hospital of Zhengzhou University, Zhengzhou, Henan 450052, China; b Department of Gastroenterology, Henan Provincial People's Hospital, Zhengzhou University People's Hospital, Zhengzhou, Henan 450004, China; c Center of Reproductive Medicine, The First Affiliated Hospital of Zhengzhou University, Zhengzhou, Henan 450052, China; d Department of Respiratory and Critical Care Medicine, The First Affiliated Hospital of Zhengzhou University, Zhengzhou, Henan 450052, China; e Department of Endovascular Surgery, The First Affiliated Hospital of Zhengzhou University, Zhengzhou, Henan 450052, China; f State Key Laboratory of Proteomics, Beijing Proteome Research Center, National Center for Protein Sciences (Beijing), Beijing Institute of Lifeomics, Beijing 102206, China; g State Key Laboratory of Medical Molecular Biology, Institute of Basic Medical Sciences, Chinese Academy of Medical Sciences, Department of Pathophysiology, Peking Union Medical College, Beijing 100730, China

Hepatocellular carcinoma (HCC) is a highly heterogeneous tumor, with dynamic equilibrium and complex interplay between its intricate tumor nature and ambient tumor immune microenvironment (TIME).^1^ Elegant research has indicated that cancer stem cells, a small subset of neoplastic cells confined within dedicated niches, display stem cell-like properties and interact with cells in TIME, thereby imparting an indelible impact on stemness regulation, tumor heterogeneity, and cancer cell plasticity.^2^ Previous taxonomies solely from the perspective of stemness or TIME may introduce some degree of bias in the comprehension of HCC carcinogenesis,^3^^,^^4^ and thus it is of paramount importance to systematically consider tumor stemness and TIME as a whole to truly portray the biological landscape of HCC.

A total of 1354 tumor patients from 11 datasets were enrolled in this study, the specifics of which were collated in Table S1. A cooperative analysis integrating stemness signatures and immune microenvironment was conducted by the iClusterBayes algorithm for HCC subtyping in The Cancer Genome Atlas (TCGA) cohort (Fig. 1A). Four stemness and immune microenvironment-based subtypes (SIS1–4) were determined to be the optimal solution for HCC according to the Bayesian information criteria and deviance ratio plots (Fig. S1A). With uniform manifold approximation and projection analysis casting HCC samples into two-dimensional spatial coordinates, evident subpopulation differentiation further verified the rationale for classifying HCC into four subtypes (Fig. S1B). Silhouette statistics further evidenced the stability and robustness of four heterogeneous subtypes, with an overwhelming majority of samples corresponding to positive silhouette widths (Fig. S1C). Furthermore, stemness profiles and immune characteristics inherent to the four subtypes were also significantly distinguished by the heatmap (Fig. S1D). To facilitate the application of SIS in the clinical context, the prognostic significance of the four subtypes was revealed with SIS4 demonstrating the poorest outcome (Fig. 1B). In summary, the four molecular subtypes were well-characterized, which shed novel light on the heterogeneity of HCC.

**Figure 1:**
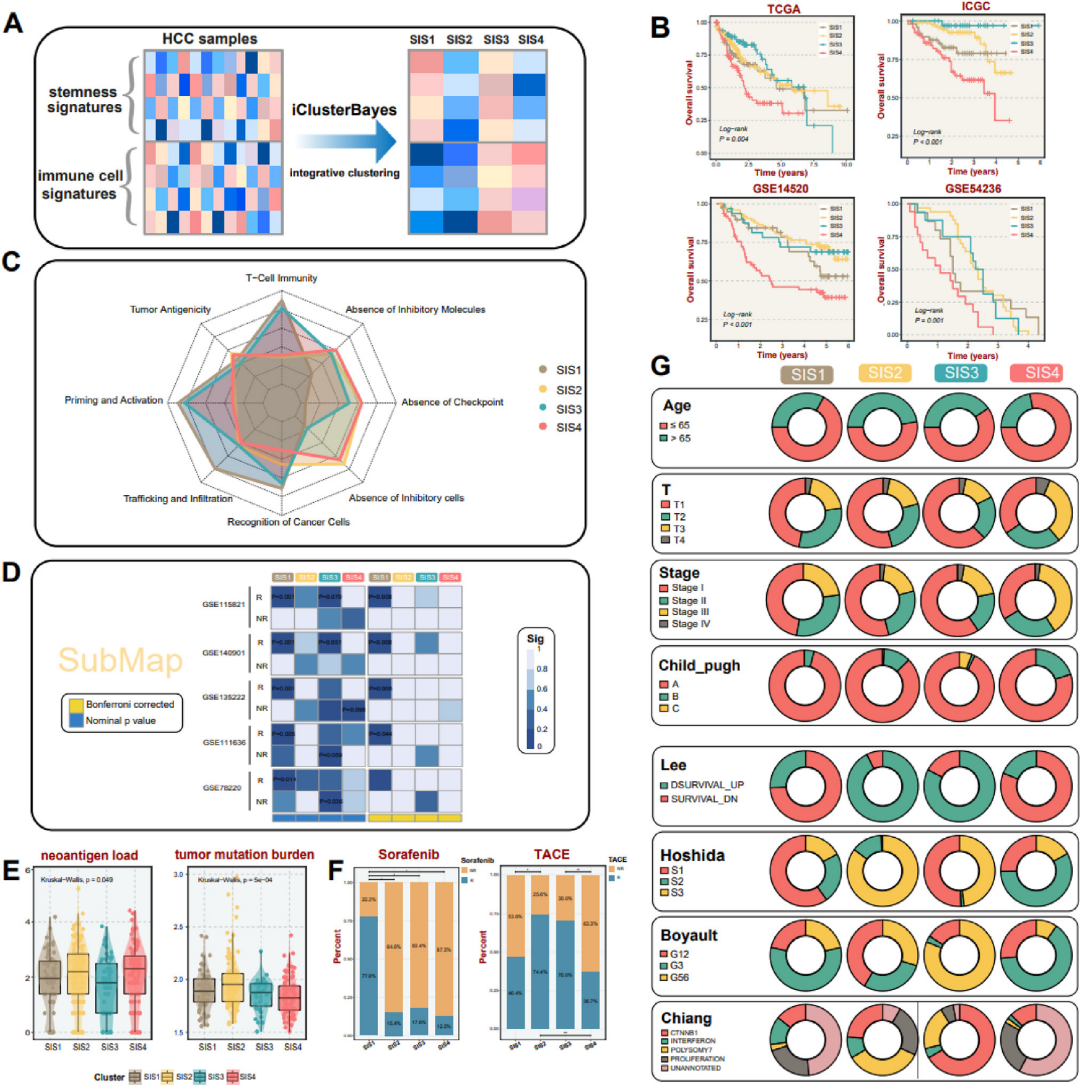
Development of HCC stemness and immune microenvironment-based subtypes (SIS) based on integrative analysis of stemness signatures and immune microenvironment. **(A)** Identification of SIS via the iClusterBayes algorithm. **(B)** Kaplan–Meier survival analysis for four SIS subtypes across TCGA cohorts and three validation cohorts including ICGC, GSE14520, and GSE54236. **(C)** Radar plot of cancer–immunity cycle of four subtypes. The circles from inner to outer represent levels from 0 to 5. **(D)** Subclass mapping analysis delineated similar gene expression patterns between TCGA and immunotherapeutic responders from five cohorts with treatment annotations. ‘*R*’ represents responder, whereas ‘NR’ represents non-responder. **(E)** Discrepancies in neoantigen load and tumor mutation burden among four subtypes (Kruskal–Wallis test). **(F)** The response rate to sorafenib and transarterial chemoembolization of four subtypes (^∗^*P* < 0.05, ^∗∗^*P* < 0.01). **(G)** The proportion of clinical metrics and several HCC molecular subtypes in our taxonomy.

To further elaborate on the robustness and reproducibility of SIS, the nearest template prediction algorithm was applied grounded on the feature genes for distinct subtypes, which were appended to Table S2. As expected, samples from three independent cohorts (ICGC, GSE14520, and GSE54236) were all confidently assigned into four clusters with SIS4 uniformly presenting the inferior survival outcome (Fig. 1B), and the expression heatmaps of feature genes for these four subtypes across three validation cohorts were demonstrated in Figure S2A–C. Fractions of four subtypes in TCGA and three validation datasets were rendered approximately equal (Fig. S2D). Subclass mapping analysis further depicted that three validation cohorts were significantly concordant with TCGA in terms of transcriptome traits (Fig. S2E). In addition, the stemness and immunological properties of the four subtypes were distinctively differentiated in all three validation datasets and were in high concordance with attributes identified in TCGA (Fig. S3).

Remarkable biological behavior disparities among heterogeneous subtypes were deciphered via gene set variation analysis with 9997 gene sets retrieved (Fig. S4A), and ontology enrichment clustering networks for distinct subtypes were also accessed with 434 prevalent gene sets through the Metascape (Fig. S4B–E). Details of the top 20 Gene Ontology biological process terms of four subtypes derived via the Metascape were summarized in Figure S5. Integrating the results of functional analysis drawn from two distinct approaches, the biological function properties of each subtype were confidently well-defined. To be more specific, SIS1 was mainly endowed with immune-activated pathways, and SIS2 demonstrated higher metabolic activity. A mixed phenotype was attributed to SIS3, considering multiple dimensions that included extracellular matrix-related pathways, extensive signaling-associated pathways, and immunogenic and stemness-related pathways presented aggregation. Characterized by pathways containing cell cycle and positive regulation of stem cell proliferation, SIS4 tended to display proliferation and stemness-related traits, echoing the worst prognosis deduced from the survival analysis.

A set of 139 molecules was examined to profile the immune landscape of the four subtypes from the perspectives of five immunomodulator groups containing antigen presentation molecules, chemokines, immunoinhibitors, immunostimulators, and receptors (Fig. S6A). Significant disparities for the four subtypes were delineated with high immunomodulator expression phenotype assigned to SIS1. The status of immunomodulator expression deficiency was also discovered in SIS2 and SIS4. Comprehensive analysis of T-cell inflammatory signature and antigen-presenting score indicated that SIS1 probably could benefit from immunotherapy with elegant performances in both two metrics (Fig. S6B, C). Cancer-immunity cycle immunogram also provided a clue for immune-activated status in SIS1, which harbored dramatically abundant immune fractions and superior tumor immunogenicity, suggesting latent immunotherapeutic potential (Fig. 1C). While SIS3 exhibited similar traits to SIS1 in many aspects such as putative existence of T-cell immunity, the absence of immune checkpoints might serve as a detrimental factor for the generation of an effective immune response in SIS3. The markedly diminished immune fractions in SIS2 and SIS4 rendered the immune-desert microenvironment in both subtypes. Moreover, dramatically elevated T cell reservation and activation were discovered in SIS1 in comparison with other subtypes (Fig. S6D–F). Common immunosuppressive cells including myeloid-derived suppressor cells, regulatory cells (Tregs), and M2 macrophages all exhibited marked infiltration patterns in SIS1 and a moderate infiltration extent in SIS3, yet minimal infiltration in SIS2 and SIS4. This pattern remained highly compatible with the abovementioned analysis (Fig. S6G–I). With the consistent performance of SIS1 in most cohorts with Bonferroni corrected *P* value < 0.05, high similarity in gene expression patterns between SIS1 samples and responders from five other immunotherapeutic cohorts was delineated via subclass mapping analysis (Fig. 1D).

As demonstrated in Figure S7A, details of the somatic mutation landscape for the four subtypes were exhibited, with significant disparities across distinct subtypes. Mutations in *TP53*, *CTNNB1*, *ALB*, and *MUSIS4* were markedly divergent among the four subtypes with *TP53* mutation significantly overrepresented in SIS1 and SIS4, *CTNNB1* and *ALB* mutations apparently enriched in SIS2, and *MUSIS4* mutation notably prevalent in SIS4 (Fig. S7B). Tight correlations between SIS4 and chromosome 1q21.3 amplification and 4q35.1 deletion, as well as close relevance of SIS1 to 8q24.21 amplification, were revealed (Fig. S7C). Additionally, SIS4 was distinguished by a higher neoantigen load, whereas SIS3 was characterized by a lower neoantigen load. SIS2 harbored a notably elevated tumor mutation burden, whereas a reduced level of tumor mutation burden was detected in SIS4 (Fig. 1E).

Correlations between each subtype and common clinical traits, as well as alignments with four published subtypes, were explored. Pronounced disparities of SIS in several prevalent clinical metrics—including age, primary tumor size, stage, and Child-Pugh classification—were recognized, along with clear differences in the link between SIS and published subtypes. As displayed in Figure 1G, SIS4 was significantly linked to advanced age, larger primary tumor size, a later stage, and Child-Pugh B cirrhosis, which might be relevant to the inferior prognosis. Intriguingly, we also observed variations in response to crucial treatment therapies such as sorafenib and transarterial chemoembolization, which acted as the backbone for improving clinical outcomes.^5^ The response rate to sorafenib was strikingly higher in SIS1, whereas the transarterial chemoembolization strategy demonstrated remarkable efficacy in SIS2 and SIS3 (Fig. 1F).

Overall, this study achieved a vital stride forward in decoding the complex interplay between tumor stemness and TIME, which augmented our perception of HCC heterogeneity and held the promise of precise patient management.

## Ethics declaration

The patient data in this work were acquired from the publicly available datasets whose informed consents of patients were complete. All authors have seen and approved the manuscript and consent publication.

## Author contributions

ZYW, SC, and DXL contributed to the study design and data analysis. SC and HX wrote and edited the manuscript. XWH, ZQL, XLL, and SYW contributed to project oversight and manuscript revisiting. YQR, YYZ, and CGG contributed to the manuscript revisiting. All authors read and approved the final manuscript.

## Conflict of interests

The authors declare that they have no competing interests.

## Data availability

Public data used in this work can be acquired from the TCGA Research Network portal (https://portal.gdc.cancer.gov/), International Cancer Genome Consortium (ICGC, https://dcc.icgc.org/), and Gene Expression Omnibus (GEO, http://www.ncbi.nlm.nih.gov/geo/).
