# Supplementary material for: Integrative analysis of tumor stemness and immune microenvironment deciphers novel molecular subtypes in hepatocellular carcinoma
Source: Genes Dis. 2023 Sep 7;11(5):101077. doi: 10.1016/j.gendis.2023.101077 (PMC11176636; doi:10.1016/j.gendis.2023.101077)
Supplement: Multimedia component 1 [file mmc1.docx]

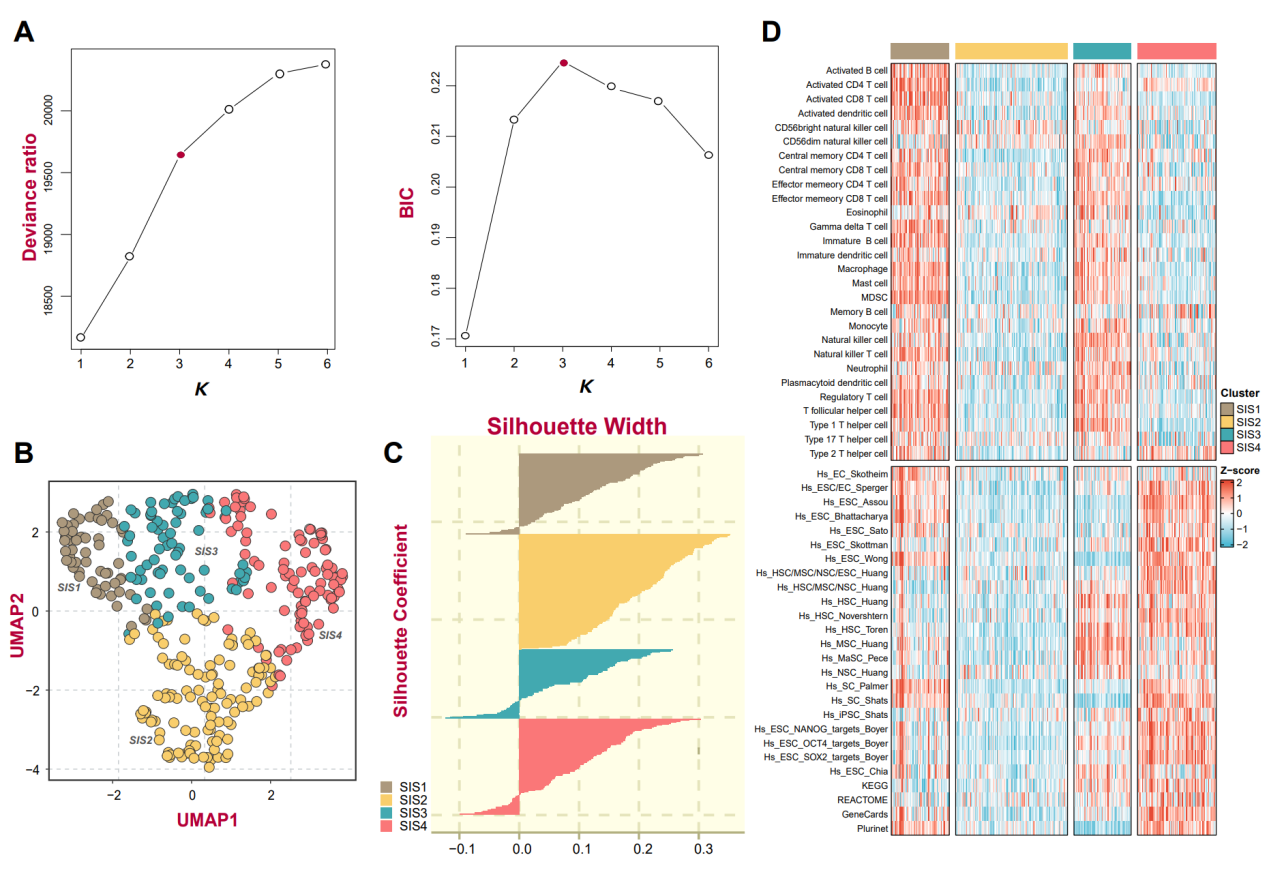


**Figure S1.** **Subtype** **identification based on integrative analysis of stemness signatures and immune microenvironment.** (A) The BIC and deviance ratio plots for determining the optimal cluster number. (B) UMAP analysis of TCGA samples. (C) Silhouette statistic of four heterogeneous subtypes. (D) Heatmap depicting the landscape of 28 immune cell infiltration and 26 stemness signatures in four subtypes.


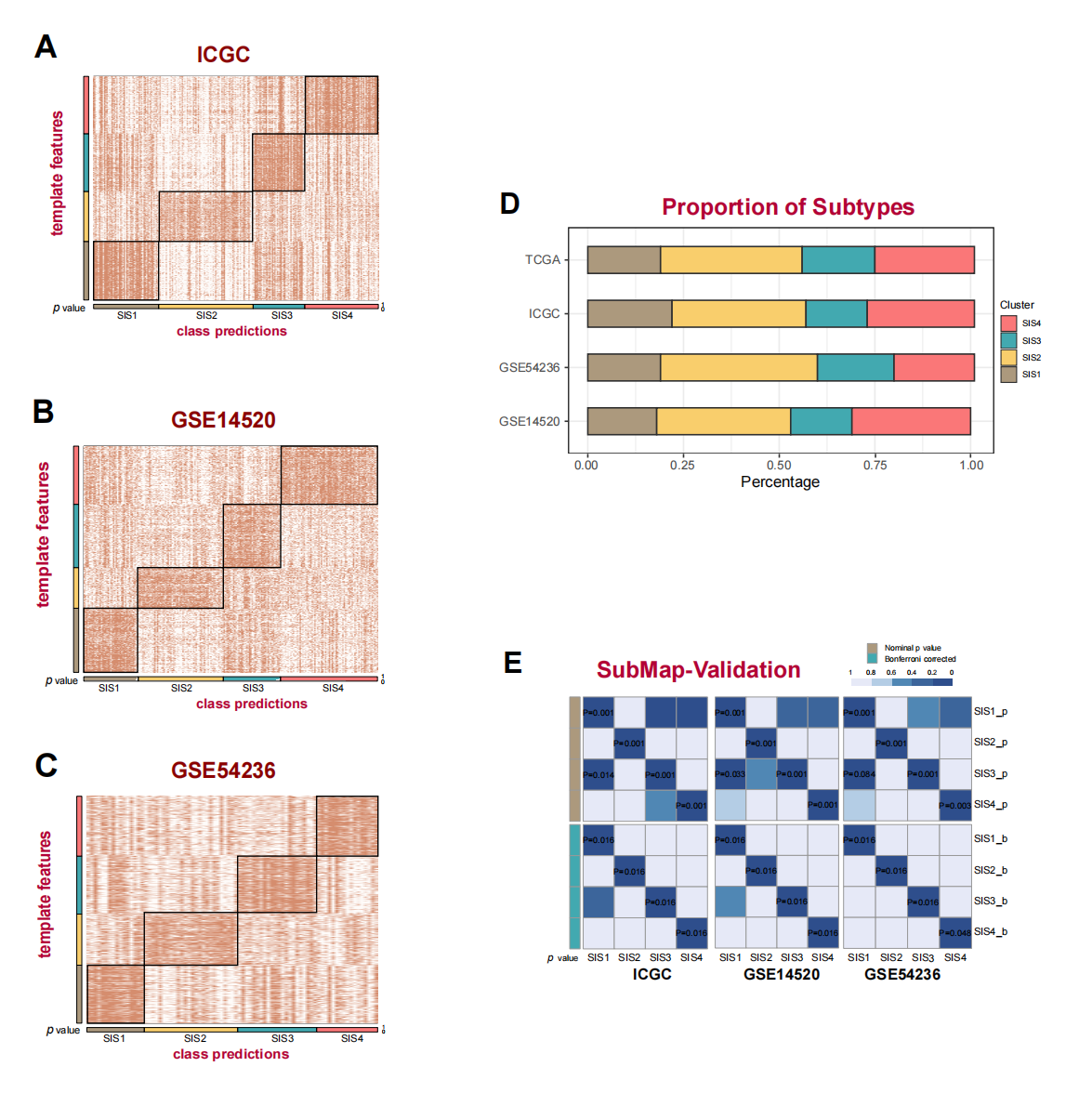
 **Figure S2**. **Validation of the robustness of HCC SIS.** (A-C) Feature genes expression heatmap for four subtypes across three validation cohorts including ICGC, GSE14520 and GSE54236. (D) The proportion of four subtypes across four cohorts. (E) SubMap analysis revealed the significant correlation of expression profiles between four subtypes across TCGA and three validation cohorts.


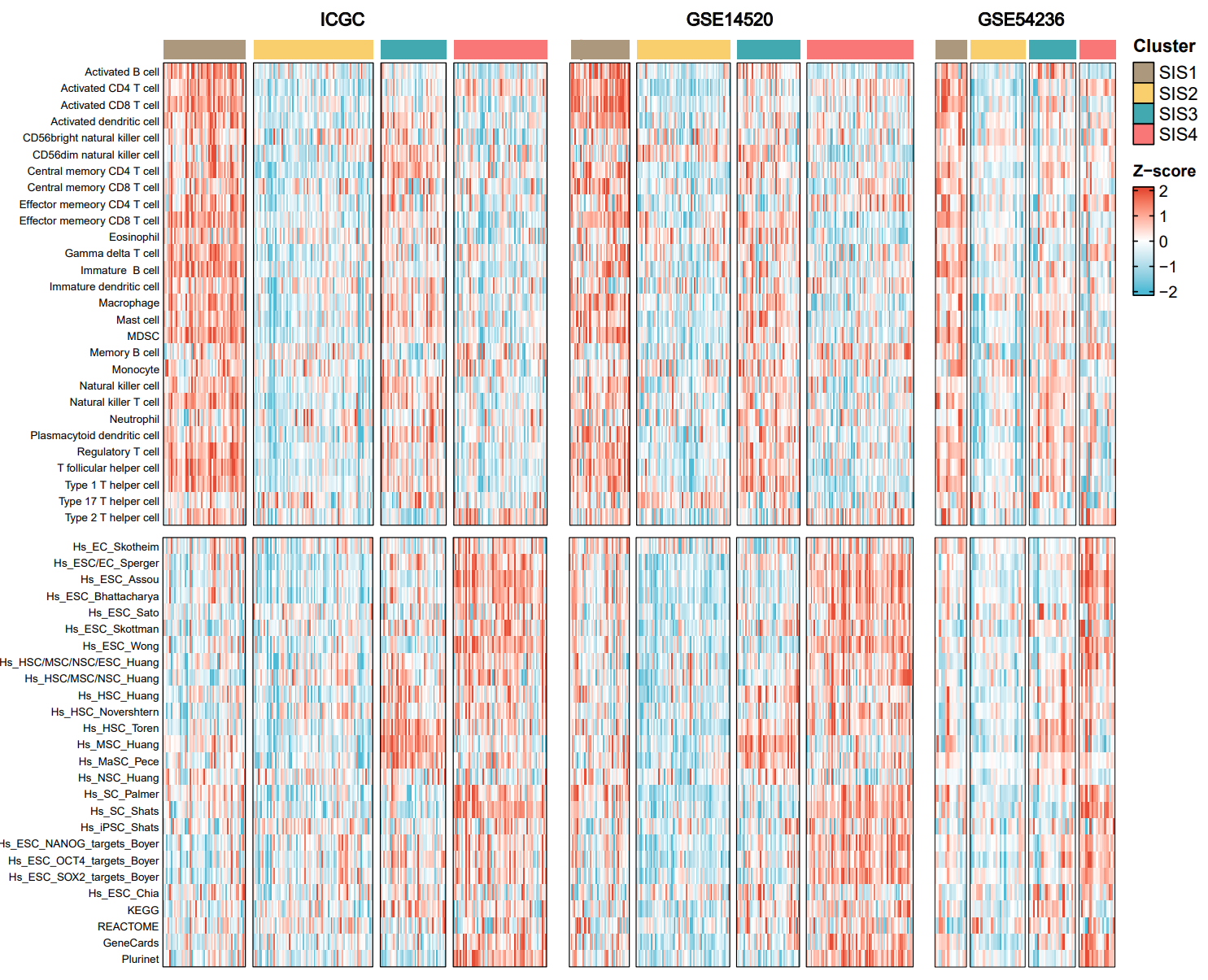


**Figure S3.** The distribution difference of 26 stemness signatures and 28 immune cell infiltration across four subtypes in ICGC, GSE14520 and GSE54236.


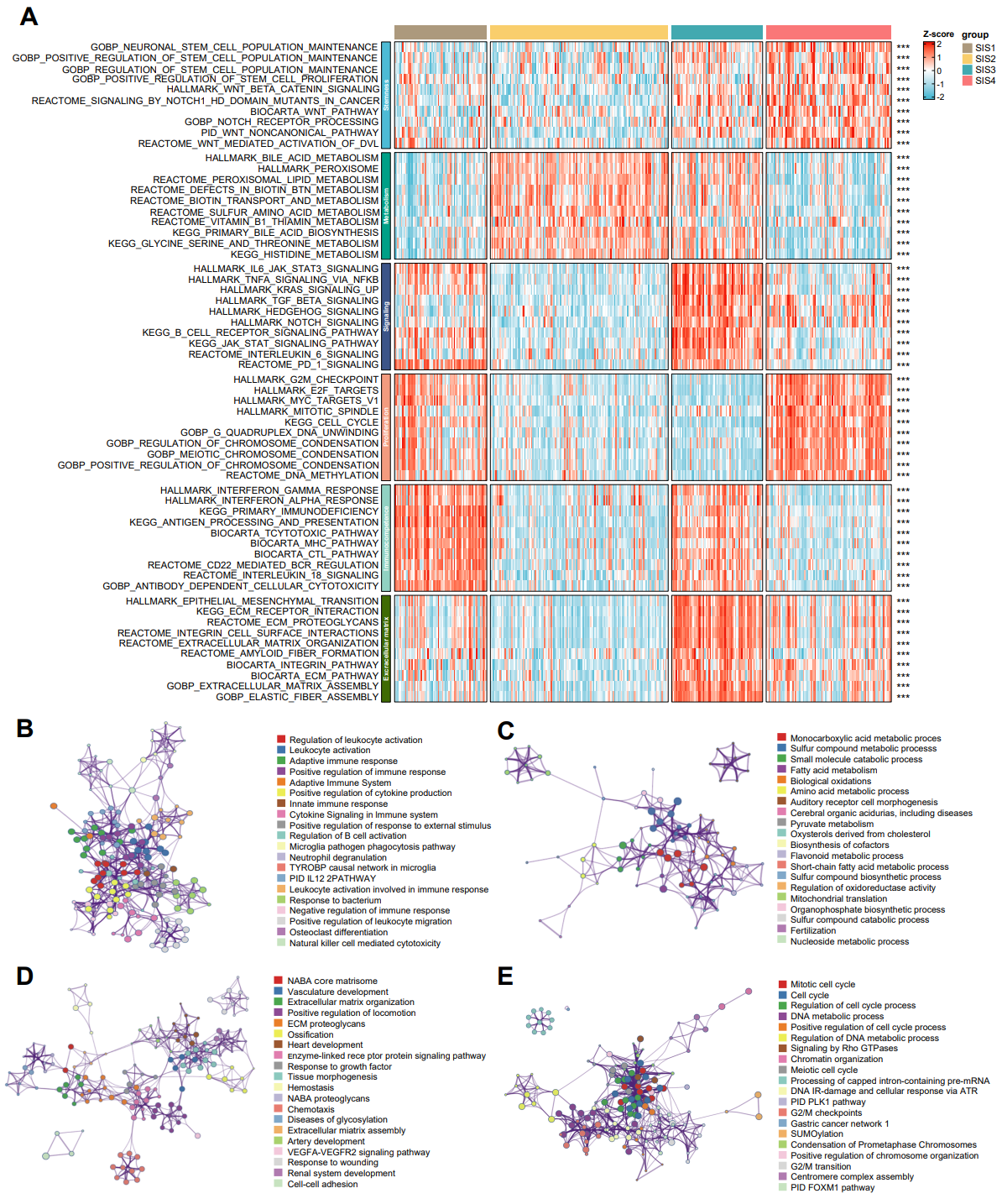


**Figure S4.** **Pathway enrichment analysis.** (A) Functional analysis for four subtypes via GSVA. (B-E) Ontology enrichment clustering networks for SIS1(B), SIS2 (C), SIS3 (D), and SIS4 (E) using the Metascape. *p <0.05, **p <0.01, ***p <0.001.


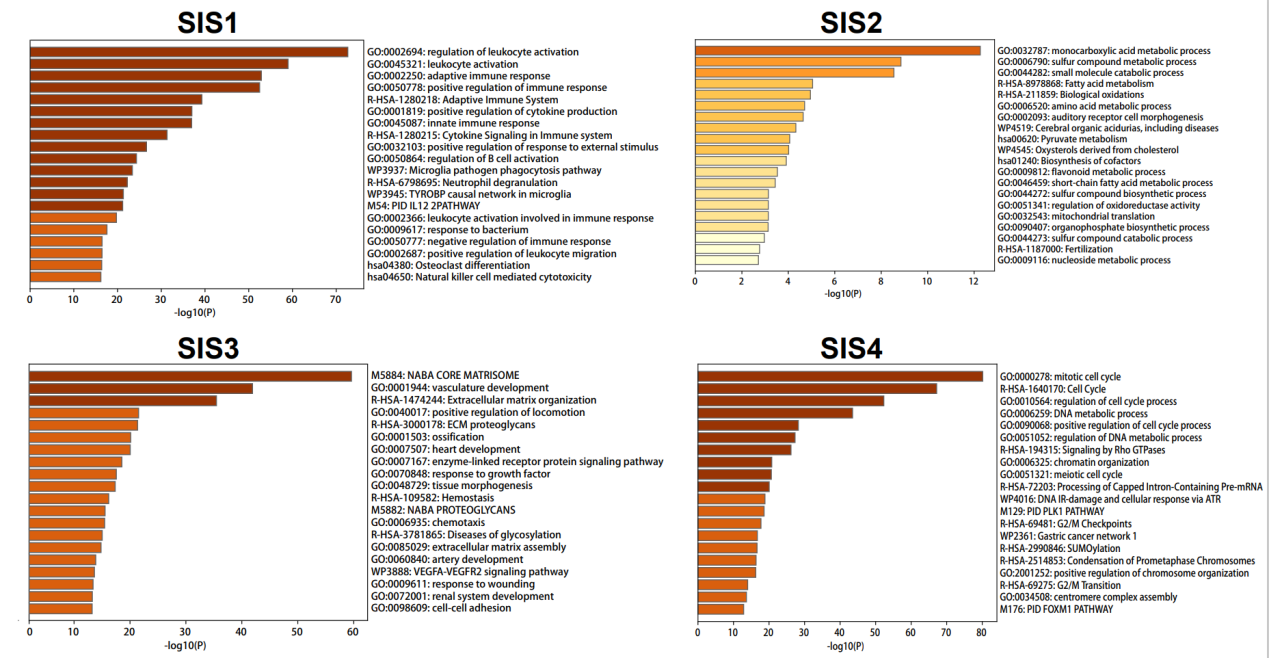


**Figure S5. Functional analysis by Metascape.** (A-D) Top 20 biological process terms of SIS1 (A), SIS2 (B), SIS3 (C), and SIS4 (D) derived via Metascape.


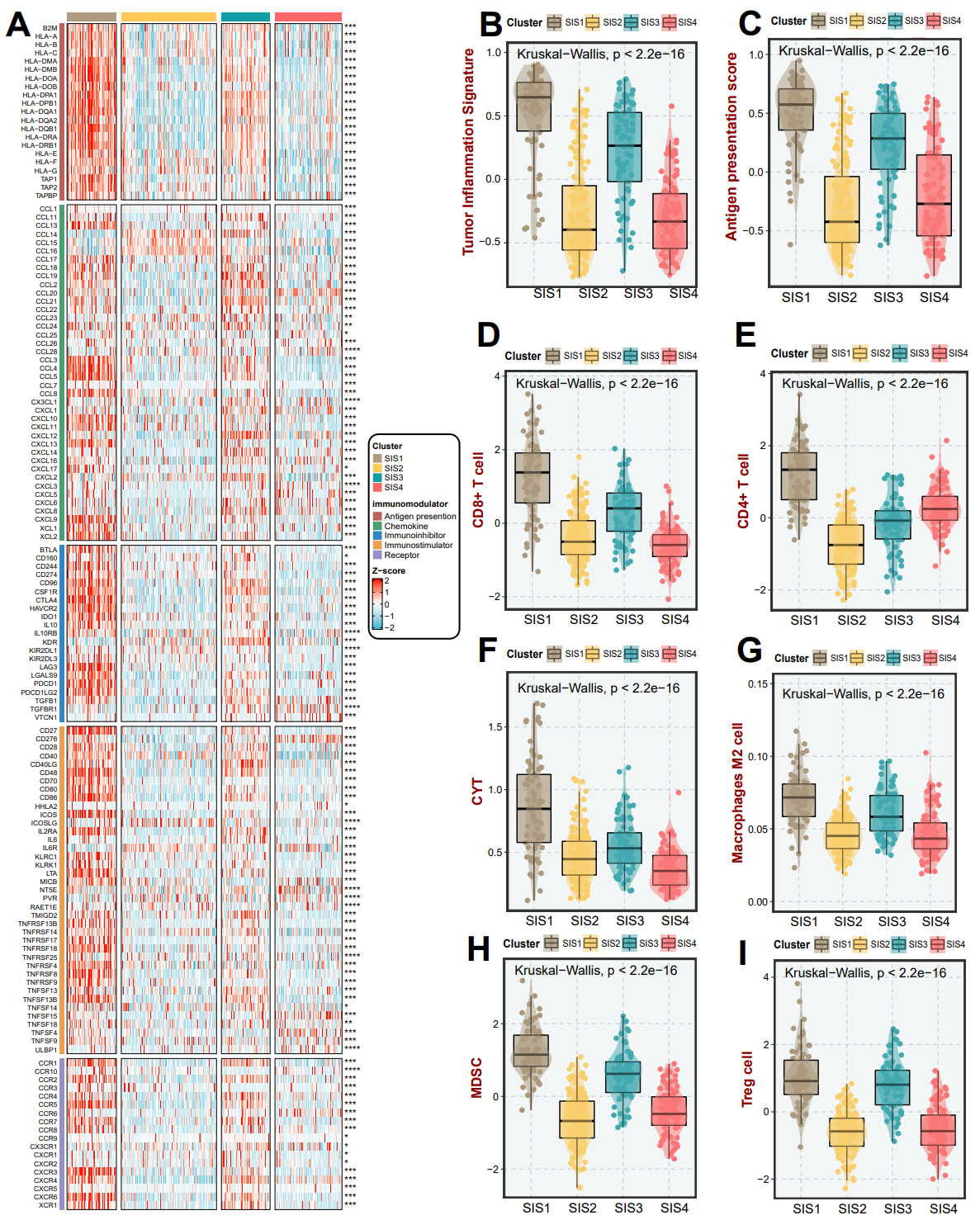


**Figure S6. Immune landscape of SIS.** (A) The expression distribution of 139 immunomodulators among six subtypes. (B-C) TIS and APS of four subtypes. (D-F) The distribution of CD4 +T cells, CD8 +T cells, and cytolytic activity (CYT) score among four subtypes via the Kruskal-Wallis test. (G-I) The distribution of MDSC, Treg cells, and macrophages M2 cells among four subtypes via the Kruskal-Wallis test. *p <0.05, **p <0.01, ***p <0.001, ****p <0.0001.


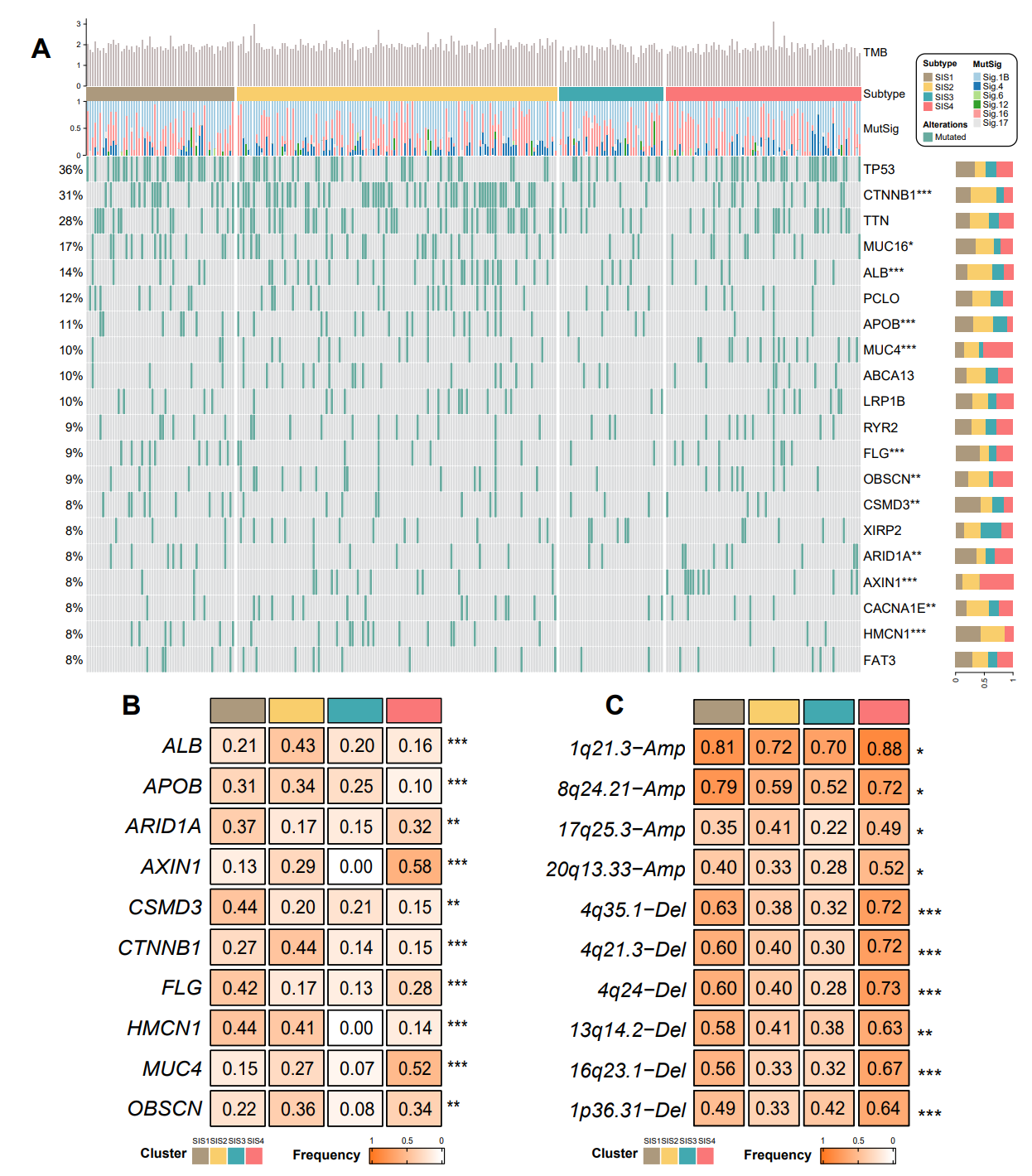


**Figure S7.** **Landscape of genomic variations.** (A) Somatic mutation landscapes of TMB, mutational signatures, and frequently mutated genes among four subtypes. (B) Comparison of top several frequently mutated genes among four subtypes. (C) Comparison of top several frequent CNV of amplification and deletion among four subtypes. *p<0.05, **p<0.01, ***p<0.001.
