# Supplementary material for: Integrative analysis of tumor stemness and immune microenvironment deciphers novel molecular subtypes in hepatocellular carcinoma
Source: Genes Dis. 2023 Sep 7;11(5):101077. doi: 10.1016/j.gendis.2023.101077 (PMC11176636; doi:10.1016/j.gendis.2023.101077)
Supplement: Multimedia component 2 [file mmc2.docx]

**Data acquisition and processing**

***Human HCC cohorts.*** A total of four independent hepatocellular carcinoma (HCC) datasets with gene expression and complete clinical details were enrolled in this study. These datasets consisted of The Cancer Genome Atlas-Liver Hepatocellular Carcinoma (TCGA-LIHC, n =369), International Cancer Genome Consortium (ICGC, n =232), Gene Expression Omnibus GSE14520 (n =247), and Gene Expression Omnibus GSE54236 (n =81). Data were obtained from TCGA (https://portal.gdc.cancer.gov/), International Cancer Genome Consortium (ICGC, https://dcc.icgc.org/), and Gene Expression Omnibus (GEO, http://www.ncbi.nlm.nih.gov/geo/). For RNA-seq data, raw count data were transformed into transcripts per million (TPM) format and subjected to further log2 conversion. Expression profiles extracted from GEO databases were processed and normalized via *affy* packages. In addition, two microarray cohorts were retrieved from GEO databases as well, encompassing GSE104580 (n =147) and GSE109211 (n = 67) with [transarterial chemoembolization (](https://pubmed.ncbi.nlm.nih.gov/28177886/)TACE) treatment and sorafenib therapy information, respectively.

***Immunotherapy cohorts.*** Five eligible datasets with immunotherapeutic annotations and expression information, consisting of GSE115821 (n =147), GSE140901 (n =24), GSE135222 (n =27), GSE111636 (n =11), and GSE78220 (n =28), were derived with next-generation sequencing performed on patients. Additional details regarding the 11 independent datasets mentioned above are surmised in supplementary table S1.

***Multi-omics data for TCGA-LIHC.*** Somatic variants were identified via TCGA VarScan2 pipeline, and copy number variation (CNV) data were acquired from FireBrowse (https://www.firebrowse.org) and processed through GISTISIS2.0.

**Subtype identification based on integrative analysis of stemness signatures and immune microenvironment**

We obtained 26 stemness signatures curated from the StemChecker webserver (<http://stemchecker.sysbiolab.eu>), where stemness signatures were well defined via multiple methods, including expression profiles, literature, computational summaries, transcription factor target genes, and RNAi screens^1^. The abundance of 28 immune cells in the tumor immune microenvironment (TIME) was evaluated by the single-sample Gene Set Enrichment Analysis (ssGSEA) algorithm with immune signatures gathered from a published study^2^. For each sample, the enrichment abundance of stemness signatures and TIME level were quantitatively determined with corresponding signatures according to the ssGSEA algorithm via *GSVA* package^3^. Subsequently, founded on gene expression profiles of stemness and immune signatures, integrative analysis for tumor stemness and immune microenvironment was employed for subtype clustering via iClusterBayes method in *iClusterPlus* package^4^. The optimum cluster number was eventually decided by the Bayesian information criteria (BIC) and deviance ratio plots between 2 and 7 clusters. Silhouette statistics were applied to evaluate the robustness of the clustering pattern by gauging how well a sample was allocated to its respective cluster. A higher silhouette value of the sample was linked to a better alignment with the cluster to which it was assigned^5^.

**Validation of the robustness of HCC subtypes**

Nearest template prediction (NTP) approach was universally applied to assess class prediction confidence for each patient. In order to ensure the dependability and reproducibility of classification, the NTP algorithm implemented in *CMScaller* package was employed in three cohorts with inconsistent platforms (ICGC, GSE14520, and GSE54236). Signature genes of each subtype for NTP analysis were listed in supplementary table S2.

To verify the accuracy of our subtypes from gene expression patterns (GEPs), the Subclass mapping (SubMap) algorithm was implemented to examine the comparability of GEPs between corresponding subtypes across different cohorts.

**Pathway enrichment analysis**

A total of 9997 gene sets utilized in this study were retrieved from Molecular Signature Database (MSigDB, https://www.gsea-msigdb.org/gsea/index.jsp) comprising Gene Ontology (GO), Kyoto Encyclopedia of Genes and Genomes (KEGG), Biocarta, hallmark, The Pathway Interaction Database (PID), and Reactome gene sets. Gene set variation analysis (GSVA) assessed pathway activity, the disparities of which across distinct subtypes were evaluated by the Kruskal-Wallis rank-sum test.

For Metascape analysis, ontology sources including GO, Reactome, KEGG, and WikiPathways gene sets^6^ were incorporated. Networks elaborated the interconnections between terms. Nodes represented functional terms, and edges signified Kappa similarities between two linked terms exceeding a score of 0.3. With term clusters represented by the most significant members, the same color indicated that corresponding members were attributed to the same term cluster.

**Evaluation of immunotherapeutic potential**

To forecast the immunotherapeutic potential of four subtypes, our study collected three metrics, comprising T-cell inflammatory signature (TIS) which consists of 18 inflammatory genes, antigen-presenting score (APS) based on 18 genes relevant to antigen processing and presenting machinery (APM), as well as cancer-immunity cycle (CIC) depicted by eight axes of the immunogram score (IGS). Of note, signatures of immunogram encompassing facets such as T cell immunity, T cell priming and activation, trafficking and infiltration of T cells into tumors, the recognition of cancer cells by T cells, inhibitory cells, checkpoint molecule expression, as well as other inhibitory molecules, were retrieved from a published study^7^. Normalized enrichment score (NES) was calculated via *GSVA* package and subsequently transformed into z-score (Z). Tumor antigenicity was assessed by neoantigen load z-score. The IGSs of T cell immunity, tumor antigenicity, T cell priming and activation, trafficking and infiltration of T cells into tumors, and the recognition of cancer cells by T cells were estimated as 3 + 1.5×Z, while the absence of inhibitory cells, absence of checkpoint molecule expression and absence of other inhibitory molecules were estimated as 3 - 1.5×Z. To further anticipate the immunotherapy response for each cluster, the SubMap algorithm was executed to explore the GEPs similarity between HCC patients and immunotherapeutic patients recruited from five immunotherapy cohorts.

**Landscape of genomic variations**

Details of distinct subtypes in somatic variants, which comprise the tumor mutation burden (TMB), mutational signatures^8^, and mutation frequency, were extracted and visualized via *maftools* package. In general, the top 20 frequently mutated genes were deemed as major driver genes for malignancies^9^. Neoantigen load (NAL) was also calculated to investigate the tumor immunogenicity of LIHC. Additionally, disparities in CNV across clusters were analyzed, focusing on regions with frequent amplifications (AMP) and homozygous deletions (HOMDEL).

**Collection of published molecular subtypes of HCC**

To uncover the latent association between our subtypes and prevalent classifications, several HCC molecular classifications with high popularity and recognition were sourced from studies conducted by Lee, Hoshida, Boyault, and Chiang et al.^10-13^.

**Statistical Analysis**

R software (version 4.12.0) was employed to process, statistically analyze and visualize data. Pearson’s chi-square test or Fisher’s exact test was utilized to analyze categorical variables, and the Kruskal-Wallis rank-sum test or T-test was performed to evaluate the variations between continuous variables. Missing clinical information from TCGA was imputed using multiple imputations with chained equations. Survival differences across subtypes were estimated by Kaplan-Meier analysis with a log-rank test via *survminer* package. A two-tailed P-value <0.05 was deemed statistically significant.

Supplementary methods reference

1. Pinto JP, Kalathur RK, Oliveira DV, et al. StemChecker: a web-based tool to discover and explore stemness signatures in gene sets. *Nucleic Acids Res.* 2015;43(W1):W72-77.

2. Charoentong P, Finotello F, Angelova M, et al. Pan-cancer Immunogenomic Analyses Reveal Genotype-Immunophenotype Relationships and Predictors of Response to Checkpoint Blockade. *Cell Rep.* 2017;18(1):248-262.

3. Hänzelmann S, Castelo R, Guinney J. GSVA: gene set variation analysis for microarray and RNA-seq data. *BMC Bioinformatics.* 2013;14:7.

4. Mo Q, Wang S, Seshan VE, et al. Pattern discovery and cancer gene identification in integrated cancer genomic data. *Proc Natl Acad Sci U S A.* 2013;110(11):4245-4250.

5. Lovmar L, Ahlford A, Jonsson M, Syvänen AC. Silhouette scores for assessment of SNP genotype clusters. *BMC Genomics.* 2005;6:35.

6. Zhou Y, Zhou B, Pache L, et al. Metascape provides a biologist-oriented resource for the analysis of systems-level datasets. *Nat Commun.* 2019;10(1):1523.

7. Karasaki T, Nagayama K, Kuwano H, et al. An Immunogram for the Cancer-Immunity Cycle: Towards Personalized Immunotherapy of Lung Cancer. *J Thorac Oncol.* 2017;12(5):791-803.

8. Alexandrov LB, Nik-Zainal S, Wedge DC, et al. Signatures of mutational processes in human cancer. *Nature.* 2013;500(7463):415-421.

9. Liu Z, Wang L, Guo C, et al. TTN/OBSCN 'Double-Hit' predicts favourable prognosis, 'immune-hot' subtype and potentially better immunotherapeutic efficacy in colorectal cancer. *J Cell Mol Med.* 2021;25(7):3239-3251.

10. Boyault S, Rickman DS, de Reyniès A, et al. Transcriptome classification of HCC is related to gene alterations and to new therapeutic targets. *Hepatology.* 2007;45(1):42-52.

11. Chiang DY, Villanueva A, Hoshida Y, et al. Focal gains of VEGFA and molecular classification of hepatocellular carcinoma. *Cancer Res.* 2008;68(16):6779-6788.

12. Hoshida Y, Nijman SM, Kobayashi M, et al. Integrative transcriptome analysis reveals common molecular subclasses of human hepatocellular carcinoma. *Cancer Res.* 2009;69(18):7385-7392.

13. Lee JS, Chu IS, Heo J, et al. Classification and prediction of survival in hepatocellular carcinoma by gene expression profiling. *Hepatology.* 2004;40(3):667-676.
