# Supplementary material for: Integrative analysis of tumor stemness and immune microenvironment deciphers novel molecular subtypes in hepatocellular carcinoma
Source: Genes Dis. 2023 Sep 7;11(5):101077. doi: 10.1016/j.gendis.2023.101077 (PMC11176636; doi:10.1016/j.gendis.2023.101077)
Supplement: Multimedia component 3 [file mmc3.docx]

**Supplementary Table S1: Details of the 11 independent datasets in this study.**

| **Accession number** | **Platform** | **Number of  patients** | **Treatment information** |
| --- | --- | --- | --- |
| TCGA-LIHC | Illumina RNAseq | 369 | No |
| ICGC-LIRI-JP | Illumina RNAseq | 232 | No |
| GSE14520 | Affymetrix Human Genome U133A Array | 221 | No |
| GSE54236 | Agilent-014850 Whole Human Genome Microarray 4x44K G4112F | 81 | No |
| GSE104580 | Affymetrix Human Genome U133 Plus 2.0 Array | 147 | TACE Treatment |
| GSE109211 | Illumina HumanHT-12 WG-DASL V4.0 expression beadchip | 67 | Sorafenib Treatment |
| GSE115821 | Illumina NextSeq 500 | 147 | Immunotherapy |
| GSE140901 | nCounter PanCancer Immune Profiling Panel | 24 | Immunotherapy |
| GSE135222 | Illumina HiSeq 2500 | 27 | Immunotherapy |
| GSE111636 | Affymetrix Human Transcriptome Array 2.0 | 11 | Immunotherapy |
| GSE78220 | Illumina HiSeq 2000 | 28 | Immunotherapy |

**Supplementary Table S2: Feature genes for distinct subtypes.**

| **class** | **gene symbol** | **class** | **gene symbol** | **class** | **gene symbol** | **class** | **gene symbol** |
| --- | --- | --- | --- | --- | --- | --- | --- |
| SIS1 | AFP | SIS2 | CYP3A4 | SIS3 | C7 | SIS4 | EPCAM |
| SIS1 | IGLL5 | SIS2 | HPD | SIS3 | LUM | SIS4 | CD24 |
| SIS1 | JCHAIN | SIS2 | CYP8B1 | SIS3 | MFAP4 | SIS4 | IGF2 |
| SIS1 | MZB1 | SIS2 | HSD11B1 | SIS3 | EFEMP1 | SIS4 | PEG10 |
| SIS1 | CCL5 | SIS2 | GLYAT | SIS3 | DCN | SIS4 | AFP |
| SIS1 | CXCL9 | SIS2 | TAT | SIS3 | CCL21 | SIS4 | VIL1 |
| SIS1 | FAM26F | SIS2 | SLC10A1 | SIS3 | AEBP1 | SIS4 | TOP2A |
| SIS1 | NKG7 | SIS2 | CYP1A1 | SIS3 | PTGDS | SIS4 | CTNND2 |
| SIS1 | MMP9 | SIS2 | AQP9 | SIS3 | CRP | SIS4 | PRR15L |
| SIS1 | CD3D | SIS2 | CYP2A6 | SIS3 | SAA2 | SIS4 | MYBL2 |
| SIS1 | HLA-DQB1 | SIS2 | SLC27A5 | SIS3 | THBS2 | SIS4 | GAL3ST1 |
| SIS1 | C1QB | SIS2 | RTP3 | SIS3 | PLA2G2A | SIS4 | DKK1 |
| SIS1 | C1QA | SIS2 | ADH4 | SIS3 | FBLN2 | SIS4 | SFRP5 |
| SIS1 | IL2RG | SIS2 | ASPDH | SIS3 | SAA1 | SIS4 | DLK1 |
| SIS1 | CXCL13 | SIS2 | TTC36 | SIS3 | C9 | SIS4 | UBE2C |
| SIS1 | PLA2G2D | SIS2 | PAGE4 | SIS3 | SLC22A1 | SIS4 | KRT19 |
| SIS1 | HLA-DRB1 | SIS2 | CYP1A2 | SIS3 | CCL19 | SIS4 | DDR1 |
| SIS1 | HLA-DQA1 | SIS2 | HPR | SIS3 | HSD17B13 | SIS4 | IGF2BP2 |
| SIS1 | CD52 | SIS2 | NOTUM | SIS3 | ISLR | SIS4 | GPC3 |
| SIS1 | SLAMF7 | SIS2 | SEC14L2 | SIS3 | COL3A1 | SIS4 | DCDC2 |
| SIS1 | CD27 | SIS2 | ALDH3A1 | SIS3 | PRELP | SIS4 | TPX2 |
| SIS1 | GZMA | SIS2 | CCL16 | SIS3 | SDS | SIS4 | WNK2 |
| SIS1 | CXCL10 | SIS2 | SLC1A2 | SIS3 | DPT | SIS4 | CDCA7 |
| SIS1 | CD2 | SIS2 | ADH1B | SIS3 | HAMP | SIS4 | FOXM1 |
| SIS1 | IL4I1 | SIS2 | RHBG | SIS3 | COL14A1 | SIS4 | SNAP25 |
| SIS1 | C1QC | SIS2 | UGT1A4 | SIS3 | PODN | SIS4 | CENPF |
| SIS1 | HLA-DPB1 | SIS2 | CYP17A1 | SIS3 | COL1A1 | SIS4 | S100A14 |
| SIS1 | CD8A | SIS2 | NR1I3 | SIS3 | CCDC80 | SIS4 | SPINT1 |
| SIS1 | CD79A | SIS2 | SERPINC1 | SIS3 | EMILIN1 | SIS4 | KIFC1 |
| SIS1 | GBP5 | SIS2 | DCXR | SIS3 | CXCL12 | SIS4 | MCM2 |
| SIS1 | HCST | SIS2 | IGSF23 | SIS3 | MMP2 | SIS4 | SOX4 |
| SIS1 | FCER1G | SIS2 | ACSM2A | SIS3 | NNMT | SIS4 | UPK3A |
| SIS1 | FCGR3A | SIS2 | ALDH1L1 | SIS3 | SAA2-SAA4 | SIS4 | SALL2 |
| SIS1 | LAG3 | SIS2 | MOGAT2 | SIS3 | BGN | SIS4 | NUSAP1 |
| SIS1 | RAC2 | SIS2 | AKR7A3 | SIS3 | INMT | SIS4 | CDC20 |
| SIS1 | SPINK1 | SIS2 | ACSM2B | SIS3 | FBLN5 | SIS4 | BEX2 |
| SIS1 | HLA-DRA | SIS2 | SULT2A1 | SIS3 | COL1A2 | SIS4 | CDK1 |
| SIS1 | LGALS2 | SIS2 | MASP2 | SIS3 | ADH1C | SIS4 | DSG2 |
| SIS1 | CD74 | SIS2 | CYP4F2 | SIS3 | CYP2A6 | SIS4 | H2AFY2 |
| SIS1 | CST7 | SIS2 | CYP2E1 | SIS3 | GGT5 | SIS4 | CCL20 |
| SIS1 | AIF1 | SIS2 | CTH | SIS3 | GEM | SIS4 | ANLN |
| SIS1 | S100A4 | SIS2 | ACSM5 | SIS3 | CRISPLD2 | SIS4 | NCK2 |
| SIS1 | CORO1A | SIS2 | CPS1 | SIS3 | ADAMTSL2 | SIS4 | ECT2 |
| SIS1 | CD8B | SIS2 | PCK1 | SIS3 | SLC25A47 | SIS4 | NEK2 |
| SIS1 | SLAMF8 | SIS2 | ADH1C | SIS3 | TAT | SIS4 | FGFR3 |
| SIS1 | CD3E | SIS2 | APOA5 | SIS3 | IGFBP5 | SIS4 | DMKN |
| SIS1 | TYROBP | SIS2 | GYS2 | SIS3 | ASPN | SIS4 | CA9 |
| SIS1 | CYBB | SIS2 | ETNPPL | SIS3 | NGFR | SIS4 | DEPDC1B |
| SIS1 | PLEK | SIS2 | PFKFB1 | SIS3 | THRSP | SIS4 | BUB1B |
| SIS1 | HLA-DOA | SIS2 | NAGS | SIS3 | TDO2 | SIS4 | KIF20A |
| SIS1 | HLA-DMA | SIS2 | UPB1 | SIS3 | MGP | SIS4 | CLGN |
| SIS1 | UCP2 | SIS2 | ACSM1 | SIS3 | VNN1 | SIS4 | CCNA2 |
| SIS1 | HLA-DMB | SIS2 | CYP2C8 | SIS3 | HSPB6 | SIS4 | MAPK13 |
| SIS1 | LAPTM5 | SIS2 | CES2 | SIS3 | COL5A1 | SIS4 | PRC1 |
| SIS1 | TMSB10 | SIS2 | REG3A | SIS3 | COL6A2 | SIS4 | NUF2 |
| SIS1 | ITGB2 | SIS2 | CYP7A1 | SIS3 | JCHAIN | SIS4 | LMNB1 |
| SIS1 | HLA-DQA2 | SIS2 | MYRIP | SIS3 | TIMP1 | SIS4 | CCNB1 |
| SIS1 | CD53 | SIS2 | DAO | SIS3 | FMOD | SIS4 | CCNB2 |
| SIS1 | CD7 | SIS2 | CLDN2 | SIS3 | SMOC2 | SIS4 | ASPM |
| SIS1 | PSMB9 | SIS2 | UGT1A3 | SIS3 | MT1G | SIS4 | MISP |
| SIS1 | CCL19 | SIS2 | SLC28A1 | SIS3 | SRPX | SIS4 | GINS1 |
| SIS1 | RGS1 | SIS2 | THRSP | SIS3 | FCN3 | SIS4 | BIRC5 |
| SIS1 | TREM2 | SIS2 | HSD17B6 | SIS3 | APLNR | SIS4 | KIF12 |
| SIS1 | HLA-DPA1 | SIS2 | AZGP1 | SIS3 | LTBP4 | SIS4 | FXYD2 |
| SIS1 | GZMH | SIS2 | ETNK2 | SIS3 | LTBP2 | SIS4 | KIF18B |
| SIS1 | CDC20 | SIS2 | SLC6A13 | SIS3 | ANTXR1 | SIS4 | MKI67 |
| SIS1 | LSP1 | SIS2 | BHMT | SIS3 | SHISA3 | SIS4 | ZWINT |
| SIS1 | PDCD1 | SIS2 | FMO3 | SIS3 | SLCO2A1 | SIS4 | PEG3 |
| SIS1 | MYBL2 | SIS2 | ABCB11 | SIS3 | LAMA2 | SIS4 | HJURP |
| SIS1 | GZMB | SIS2 | GNMT | SIS3 | SOD3 | SIS4 | DTL |
| SIS1 | DOK2 | SIS2 | C1QTNF3 | SIS3 | ELN | SIS4 | PTK7 |
| SIS1 | HLA-DQB2 | SIS2 | CYP2D6 | SIS3 | ADAMTS2 | SIS4 | TYMS |
| SIS1 | SELPLG | SIS2 | PRAP1 | SIS3 | FOS | SIS4 | LRRC1 |
| SIS1 | CCL4 | SIS2 | CYP2C9 | SIS3 | OGN | SIS4 | SOX9 |
| SIS1 | BATF | SIS2 | CDO1 | SIS3 | ADH4 | SIS4 | BICC1 |
| SIS1 | COTL1 | SIS2 | SRD5A2 | SIS3 | PTGIS | SIS4 | FGFR2 |
| SIS1 | SPHK1 | SIS2 | SLC22A1 | SIS3 | EGR1 | SIS4 | FOXQ1 |
| SIS1 | FOLR2 | SIS2 | CYP2A7 | SIS3 | MFSD2A | SIS4 | SMC4 |
| SIS1 | CAPG | SIS2 | LDHD | SIS3 | HSPG2 | SIS4 | PBK |
| SIS1 | BCL2A1 | SIS2 | F12 | SIS3 | MT1E | SIS4 | LAPTM4B |
| SIS1 | LST1 | SIS2 | OTC | SIS3 | CFHR4 | SIS4 | PDX1 |
| SIS1 | HLA-DRB5 | SIS2 | OSGIN1 | SIS3 | APOA4 | SIS4 | SUSD4 |
| SIS1 | SASH3 | SIS2 | FETUB | SIS3 | F9 | SIS4 | FRAS1 |
| SIS1 | RGS10 | SIS2 | GLYATL1 | SIS3 | CLEC3B | SIS4 | DUSP9 |
| SIS1 | PRF1 | SIS2 | CPN2 | SIS3 | C1QTNF1 | SIS4 | PLK1 |
| SIS1 | S100P | SIS2 | SLC13A3 | SIS3 | PRG4 | SIS4 | NPNT |
| SIS1 | CD48 | SIS2 | UBXN10 | SIS3 | IGFBP7 | SIS4 | COLCA2 |
| SIS1 | SPI1 | SIS2 | ABCB4 | SIS3 | SOCS3 | SIS4 | ANXA13 |
| SIS1 | FERMT3 | SIS2 | GREM2 | SIS3 | CYP2C8 | SIS4 | GTSE1 |
| SIS1 | LCP1 | SIS2 | HP | SIS3 | HGFAC | SIS4 | MELK |
| SIS1 | HCK | SIS2 | UGT2B10 | SIS3 | EMP1 | SIS4 | TROAP |
| SIS1 | LCK | SIS2 | AMACR | SIS3 | MT1H | SIS4 | PPAP2C |
| SIS1 | PTTG1 | SIS2 | PON1 | SIS3 | LRRC32 | SIS4 | PLEKHB1 |
| SIS1 | VNN2 | SIS2 | RDH16 | SIS3 | THBD | SIS4 | ARID3A |
| SIS1 | HLA-DOB | SIS2 | AR | SIS3 | CCL2 | SIS4 | ELOVL7 |
| SIS1 | ANKRD22 | SIS2 | HLF | SIS3 | STEAP4 | SIS4 | CDCA8 |
| SIS1 | CTSW | SIS2 | DMGDH | SIS3 | TAGLN | SIS4 | NEURL3 |
| SIS1 | ALOX5AP | SIS2 | HFE2 | SIS3 | VCAN | SIS4 | MAL2 |
| SIS1 | SIRPG | SIS2 | ENPP7 | SIS3 | HPGD | SIS4 | NT5DC2 |
| SIS1 | LTB | SIS2 | GPT2 | SIS3 | TEK | SIS4 | IGF2BP1 |
| SIS1 | RARRES3 | SIS2 | AGXT | SIS3 | SLC1A1 | SIS4 | NID1 |
| SIS1 | TNFAIP8L2 | SIS2 | CYP4A22 | SIS3 | AKR7A3 | SIS4 | KIF23 |
| SIS1 | ETV7 | SIS2 | SLC51A | SIS3 | C11orf96 | SIS4 | PYCR1 |
| SIS1 | UBD | SIS2 | TSKU | SIS3 | COLEC11 | SIS4 | TCEAL8 |
| SIS1 | CXCR3 | SIS2 | SPDYC | SIS3 | C6 | SIS4 | ITM2C |
| SIS1 | GPSM3 | SIS2 | DSG1 | SIS3 | RCAN2 | SIS4 | RAB25 |
| SIS1 | S100A9 | SIS2 | CUX2 | SIS3 | FOSB | SIS4 | TUBA1B |
| SIS1 | PYCARD | SIS2 | APOC3 | SIS3 | MT2A | SIS4 | NCAPH |
| SIS1 | SIT1 | SIS2 | SLC22A11 | SIS3 | THBS1 | SIS4 | MSI1 |
| SIS1 | RNASE6 | SIS2 | NAT2 | SIS3 | HAO2 | SIS4 | AURKB |
| SIS1 | IGSF6 | SIS2 | FNDC5 | SIS3 | COL6A1 | SIS4 | QSOX1 |
| SIS1 | CD72 | SIS2 | CFHR4 | SIS3 | RAMP3 | SIS4 | TMC4 |
| SIS1 | NCF4 | SIS2 | HAO1 | SIS3 | HRG | SIS4 | DLGAP5 |
| SIS1 | GZMK | SIS2 | AOX1 | SIS3 | CXCL14 | SIS4 | E2F1 |
| SIS1 | DUSP2 | SIS2 | SLC6A12 | SIS3 | ADAMTS1 | SIS4 | NCAPG |
| SIS1 | CPVL | SIS2 | MME | SIS3 | SLC10A1 | SIS4 | KIF2C |
| SIS1 | TRIM31 | SIS2 | TM6SF2 | SIS3 | ENG | SIS4 | TTK |
| SIS1 | LY96 | SIS2 | ACADL | SIS3 | MT1X | SIS4 | MCM3 |
| SIS1 | PLTP | SIS2 | IL27 | SIS3 | PDGFRA | SIS4 | SLC34A2 |
| SIS1 | EBI3 | SIS2 | GPT | SIS3 | F2R | SIS4 | UHRF1 |
| SIS1 | JAK3 | SIS2 | PCK2 | SIS3 | HSD11B1 | SIS4 | ATP1A1 |
| SIS1 | SPP1 | SIS2 | SLC13A5 | SIS3 | IL7R | SIS4 | ZIC2 |
| SIS1 | LILRB4 | SIS2 | RIPPLY1 | SIS3 | STMN2 | SIS4 | CDC6 |
| SIS1 | GPNMB | SIS2 | C8A | SIS3 | LHFP | SIS4 | KIF4A |
| SIS1 | CCL3 | SIS2 | TBX3 | SIS3 | CCDC3 | SIS4 | EPS8L3 |
| SIS1 | CCL4L2 | SIS2 | F9 | SIS3 | CYBRD1 | SIS4 | NREP |
| SIS1 | SRGN | SIS2 | PEX11G | SIS3 | MYH11 | SIS4 | SGCE |
| SIS1 | EVI2B | SIS2 | CDHR5 | SIS3 | COLEC10 | SIS4 | CDT1 |
| SIS1 | BIRC5 | SIS2 | NECAB2 | SIS3 | TMEM119 | SIS4 | ERICH5 |
| SIS1 | HAPLN3 | SIS2 | FMO4 | SIS3 | POSTN | SIS4 | LDOC1 |
| SIS1 | SH2D2A | SIS2 | SEPT4 | SIS3 | GYS2 | SIS4 | ESRP1 |
| SIS1 | MMP12 | SIS2 | ATP6V0E2 | SIS3 | EEF1A2 | SIS4 | SLC29A4 |
| SIS1 | DEF6 | SIS2 | AASS | SIS3 | ITGBL1 | SIS4 | KIF11 |
| SIS1 | PTPN7 | SIS2 | SULT1B1 | SIS3 | PLAT | SIS4 | ASF1B |
| SIS1 | APOBEC3C | SIS2 | TM7SF2 | SIS3 | PCK1 | SIS4 | MCM6 |
| SIS1 | PLA2G7 | SIS2 | CYP4A11 | SIS3 | ASPG | SIS4 | UBE2T |
| SIS1 | CTLA4 | SIS2 | RASL10B | SIS3 | IL33 | SIS4 | C1orf106 |
| SIS1 | GMFG | SIS2 | HGD | SIS3 | LAMC3 | SIS4 | MCM4 |
| SIS1 | AURKB | SIS2 | EHHADH | SIS3 | KCTD12 | SIS4 | DBN1 |
| SIS1 | HAVCR2 | SIS2 | TGFBR3L | SIS3 | AQP1 | SIS4 | NGFRAP1 |
| SIS1 | IL18 | SIS2 | PIPOX | SIS3 | HAND2 | SIS4 | CDKN3 |
| SIS1 | TNFRSF18 | SIS2 | ODAM | SIS3 | CFHR3 | SIS4 | RAD51AP1 |
| SIS1 | SLC1A5 | SIS2 | CYP4F11 | SIS3 | ADGRA2 | SIS4 | BMF |
| SIS1 | PLEKHO1 | SIS2 | SLC22A12 | SIS3 | FSTL1 | SIS4 | EVC |
| SIS1 | LIMD2 | SIS2 | OGDHL | SIS3 | CRHBP | SIS4 | RACGAP1 |
| SIS1 | CXCL11 | SIS2 | CHAD | SIS3 | MYL9 | SIS4 | CTSV |
| SIS1 | FCGR1A | SIS2 | SP5 | SIS3 | FBN1 | SIS4 | PTTG1 |
| SIS1 | EMP3 | SIS2 | SLCO1B3 | SIS3 | C8orf4 | SIS4 | TRIP13 |
| SIS1 | LY86 | SIS2 | BAAT | SIS3 | GAS6 | SIS4 | KCTD17 |
| SIS1 | ARHGAP9 | SIS2 | APOF | SIS3 | ALPL | SIS4 | BUB1 |
| SIS1 | HCLS1 | SIS2 | ABCG2 | SIS3 | SNAI1 | SIS4 | ATAD2 |
| SIS1 | UBE2C | SIS2 | NKD1 | SIS3 | LOXL1 | SIS4 | EHF |
| SIS1 | CD37 | SIS2 | SLC47A1 | SIS3 | MOXD1 | SIS4 | PKM |
| SIS1 | IFI27L2 | SIS2 | GFRA1 | SIS3 | MT1M | SIS4 | LAMC1 |
| SIS1 | HK3 | SIS2 | NR1I2 | SIS3 | SVEP1 | SIS4 | CDCA5 |
| SIS1 | VAV1 | SIS2 | CES1 | SIS3 | ECM1 | SIS4 | TMEM132A |
| SIS1 | MDK | SIS2 | GCAT | SIS3 | UGT1A4 | SIS4 | GRB7 |
| SIS1 | G6PD | SIS2 | RAMP1 | SIS3 | BASP1 | SIS4 | PRAME |
| SIS1 | CD6 | SIS2 | ECM2 | SIS3 | COL6A3 | SIS4 | TCF19 |
| SIS1 | LGALS9 | SIS2 | SPP2 | SIS3 | EPHA3 | SIS4 | KIF15 |
| SIS1 | CSF1R | SIS2 | APOA2 | SIS3 | GPD1 | SIS4 | RRM2 |
| SIS1 | CD86 | SIS2 | AFM | SIS3 | CYP3A4 | SIS4 | ZNF496 |
| SIS1 | WAS | SIS2 | APOC4 | SIS3 | HEYL | SIS4 | NRSN2 |
| SIS1 | TAP1 | SIS2 | ADH6 | SIS3 | CHI3L1 | SIS4 | C12orf75 |
| SIS1 | SELM | SIS2 | ABCA6 | SIS3 | PMP22 | SIS4 | MARCKSL1 |
| SIS1 | PTGDS | SIS2 | G6PC | SIS3 | CPXM2 | SIS4 | FOXJ1 |
| SIS1 | CYTH4 | SIS2 | CHADL | SIS3 | MXRA8 | SIS4 | MEP1A |
| SIS1 | C1orf162 | SIS2 | RORC | SIS3 | SULF2 | SIS4 | CKAP2L |
| SIS1 | CECR1 | SIS2 | TTR | SIS3 | MRC1 | SIS4 | CEP55 |
| SIS1 | CARD16 | SIS2 | CYP11A1 | SIS3 | TTC36 | SIS4 | GYLTL1B |
| SIS1 | C3AR1 | SIS2 | ANGPTL3 | SIS3 | FAM163B | SIS4 | PRR11 |
| SIS1 | PSTPIP1 | SIS2 | DPYS | SIS3 | COL15A1 | SIS4 | SAPCD2 |
| SIS1 | MS4A6A | SIS2 | DEPDC7 | SIS3 | PDZK1IP1 | SIS4 | MMP11 |
| SIS1 | GZMM | SIS2 | RBP4 | SIS3 | FSTL3 | SIS4 | LAD1 |
| SIS1 | HLA-B | SIS2 | RUNDC3B | SIS3 | UROC1 | SIS4 | ITGB1 |
| SIS1 | ADAMDEC1 | SIS2 | TMEM82 | SIS3 | PLTP | SIS4 | ACTG1 |
| SIS1 | S100A11 | SIS2 | CES4A | SIS3 | DNASE1L3 | SIS4 | CLDN1 |
| SIS1 | AOAH | SIS2 | EVA1A | SIS3 | NAT2 | SIS4 | SKA3 |
| SIS1 | SLAMF6 | SIS2 | DHRS2 | SIS3 | GLYATL1 | SIS4 | CENPA |
| SIS1 | NNMT | SIS2 | KNG1 | SIS3 | IFITM1 | SIS4 | HUNK |
| SIS1 | CCR5 | SIS2 | EPHX1 | SIS3 | TNXB | SIS4 | KLF5 |
| SIS1 | ARHGAP30 | SIS2 | LEPR | SIS3 | TMEM204 | SIS4 | ITIH5 |
| SIS1 | LAIR1 | SIS2 | MAT1A | SIS3 | DEFB1 | SIS4 | NRM |
| SIS1 | IL18BP | SIS2 | ADHFE1 | SIS3 | CALCRL | SIS4 | MARCKS |
| SIS1 | TIGIT | SIS2 | CYP39A1 | SIS3 | ITGA9 | SIS4 | E2F8 |
| SIS1 | CD163 | SIS2 | FBXO31 | SIS3 | SFRP4 | SIS4 | SPHK1 |
| SIS1 | CYBA | SIS2 | CYP2B6 | SIS3 | ACKR1 | SIS4 | HACD2 |
| SIS1 | CXCR6 | SIS2 | DIO1 | SIS3 | ETS1 | SIS4 | PITX1 |
| SIS1 | CYTIP | SIS2 | HEPACAM | SIS3 | CYR61 | SIS4 | ABHD3 |
| SIS1 | CENPM | SIS2 | CES3 | SIS3 | SLC46A3 | SIS4 | B3GNT3 |
| SIS1 | NCKAP1L | SIS2 | CYP4F3 | SIS3 | TIMP2 | SIS4 | EXO1 |
| SIS1 | S100A6 | SIS2 | SLC16A11 | SIS3 | FIBIN | SIS4 | GMNN |
| SIS1 | CD300A | SIS2 | ACSS3 | SIS3 | ANGPTL1 | SIS4 | LAMB1 |
| SIS1 | DUSP9 | SIS2 | FBXO2 | SIS3 | CXCL2 | SIS4 | SPINT2 |
| SIS1 | RGS2 | SIS2 | C6 | SIS3 | CLEC11A | SIS4 | HMGA1 |
| SIS1 | KIF2C | SIS2 | MTHFD1 | SIS3 | CTSK | SIS4 | B4GALNT4 |
| SIS1 | COX7B2 | SIS2 | KCNJ8 | SIS3 | UGT2B15 | SIS4 | COL2A1 |
| SIS1 | CRP | SIS2 | CA5A | SIS3 | CTGF | SIS4 | TRNP1 |
| SIS1 | MAP4K1 | SIS2 | ABHD1 | SIS3 | F13A1 | SIS4 | CENPW |
| SIS1 | FPR1 | SIS2 | RDH5 | SIS3 | GHR | SIS4 | SLC44A3 |
| SIS1 | BASP1 | SIS2 | SHMT1 | SIS3 | OGDHL | SIS4 | C1orf116 |
| SIS1 | THEMIS2 | SIS2 | PROZ | SIS3 | ADH1B | SIS4 | LRIG3 |
| SIS1 | PCED1B | SIS2 | GPD1 | SIS3 | MBL2 | SIS4 | SMC3 |
| SIS1 | RENBP | SIS2 | SELENBP1 | SIS3 | NOTCH3 | SIS4 | CCNE1 |
| SIS1 | IL10RA | SIS2 | ACY3 | SIS3 | TMEM173 | SIS4 | FAM60A |
| SIS1 | MS4A4A | SIS2 | RBP5 | SIS3 | RDH16 | SIS4 | EEF1A1 |
| SIS1 | CD5L | SIS2 | ECHDC3 | SIS3 | CD93 | SIS4 | NCAPD2 |
| SIS1 | TIMP1 | SIS2 | CYP4F12 | SIS3 | LBP | SIS4 | RPS12 |
| SIS1 | PILRA | SIS2 | ANKRD24 | SIS3 | MRC2 | SIS4 | ZBTB12 |
| SIS1 | PIK3CD | SIS2 | PAH | SIS3 | FNDC1 | SIS4 | BCAM |
| SIS1 | TNFSF13B | SIS2 | AADAC | SIS3 | ETNPPL | SIS4 | WBP5 |
| SIS1 | VCAM1 | SIS2 | CLDN14 | SIS3 | CYP39A1 | SIS4 | CHAF1B |
| SIS1 | CLEC2B | SIS2 | REN | SIS3 | SPON1 | SIS4 | ARHGAP11A |
| SIS1 | CCNB1 | SIS2 | SORD | SIS3 | MAP3K5 | SIS4 | NDRG1 |
| SIS1 | FGL2 | SIS2 | AKR1C4 | SIS3 | LDB2 | SIS4 | KPNA2 |
| SIS1 | IDO1 | SIS2 | ASPSCR1 | SIS3 | COL4A2 | SIS4 | PM20D2 |
| SIS1 | WARS | SIS2 | ST3GAL6 | SIS3 | APOF | SIS4 | FANCI |
| SIS1 | CD300LF | SIS2 | ADI1 | SIS3 | UGT1A1 | SIS4 | MCM10 |
| SIS1 | FABP5 | SIS2 | RGN | SIS3 | ID1 | SIS4 | FMO1 |
| SIS1 | LRRC25 | SIS2 | ANG | SIS3 | ANXA1 | SIS4 | ACSL4 |
| SIS1 | FYB | SIS2 | FTCD | SIS3 | DPYSL3 | SIS4 | KIF14 |
| SIS1 | S1PR4 | SIS2 | KCNB1 | SIS3 | ACSM5 | SIS4 | FAM3B |
| SIS1 | FDCSP | SIS2 | PRODH | SIS3 | VSIG4 | SIS4 | BCL9 |
| SIS1 | XCL2 | SIS2 | ABAT | SIS3 | HLA-DPA1 | SIS4 | DQX1 |
| SIS1 | MARCO | SIS2 | ALDH6A1 | SIS3 | ANO1 | SIS4 | PDE9A |
| SIS1 | AIM2 | SIS2 | LECT2 | SIS3 | ADRA2A | SIS4 | SALL4 |
| SIS1 | CTSC | SIS2 | ANXA10 | SIS3 | CYP1A2 | SIS4 | SCTR |
| SIS1 | PLA2G2A | SIS2 | UGT2B7 | SIS3 | CYP2A7 | SIS4 | HMGB2 |
| SIS1 | SIGLEC1 | SIS2 | SLC6A2 | SIS3 | HAL | SIS4 | TMEM246 |
| SIS1 | MPEG1 | SIS2 | SAA4 | SIS3 | CDH5 | SIS4 | MFI2 |
| SIS1 | TMSB4X | SIS2 | GSTA1 | SIS3 | PDGFRB | SIS4 | CKAP4 |
| SIS1 | GBP4 | SIS2 | PPP1R3G | SIS3 | COL4A1 | SIS4 | RAD54L |
| SIS1 | CCL18 | SIS2 | MACROD1 | SIS3 | GAS1 | SIS4 | CENPM |
| SIS1 | SLA | SIS2 | PLIN5 | SIS3 | FLNA | SIS4 | KNTC1 |
| SIS1 | CFD | SIS2 | GPAM | SIS3 | SPARC | SIS4 | GLIS2 |
| SIS1 | LCN2 | SIS2 | F11 | SIS3 | FRZB | SIS4 | OIP5 |
| SIS1 | VSIG4 | SIS2 | HRG | SIS3 | ACTA2 | SIS4 | MLK4 |
| SIS1 | SLC15A3 | SIS2 | ZMYND12 | SIS3 | SERPINE1 | SIS4 | MCM7 |
| SIS1 | ARHGDIB | SIS2 | CFHR5 | SIS3 | CP | SIS4 | DEPDC1 |
| SIS1 | SAMSN1 | SIS2 | GLUL | SIS3 | SUSD2 | SIS4 | ITPR3 |
| SIS1 | IL2RB | SIS2 | RP5-966M1.6 | SIS3 | RAI2 | SIS4 | SPIN1 |
| SIS1 | TMC8 | SIS2 | ACBD4 | SIS3 | MT1F | SIS4 | HMMR |
| SIS1 | CTSS | SIS2 | PDK4 | SIS3 | RASD1 | SIS4 | TESC |
| SIS1 | C15orf48 | SIS2 | ACSL5 | SIS3 | MSC | SIS4 | SERPINH1 |
| SIS1 | PKM | SIS2 | GCGR | SIS3 | KDR | SIS4 | CKS2 |
| SIS1 | AGR2 | SIS2 | MTTP | SIS3 | BCO2 | SIS4 | ETV4 |
| SIS1 | PTAFR | SIS2 | HIST1H2AE | SIS3 | AVPR1A | SIS4 | RAB3D |
| SIS1 | SH2D1A | SIS2 | PON3 | SIS3 | TXNIP | SIS4 | IGSF3 |
| SIS1 | ACP5 | SIS2 | ZNF385B | SIS3 | ID4 | SIS4 | STK26 |
| SIS1 | CMKLR1 | SIS2 | GADD45G | SIS3 | FILIP1L | SIS4 | YWHAQ |
| SIS1 | CXCR4 | SIS2 | NUGGC | SIS3 | SLPI | SIS4 | PAFAH1B3 |
| SIS1 | CD5 | SIS2 | CYP2A13 | SIS3 | LXN | SIS4 | TRIM71 |
| SIS1 | RRM2 | SIS2 | SLC22A18 | SIS3 | ZFP36 | SIS4 | PSPH |
| SIS1 | APOBEC3G | SIS2 | GPLD1 | SIS3 | CD163 | SIS4 | MAD2L1 |
| SIS1 | PIM2 | SIS2 | TTC9 | SIS3 | MYOF | SIS4 | CLIC1 |
| SIS1 | GPC3 | SIS2 | EEF1A2 | SIS3 | EDNRB | SIS4 | NDC80 |
| SIS1 | SLC7A7 | SIS2 | C4BPB | SIS3 | SRGN | SIS4 | CBX1 |
| SIS1 | CCNB2 | SIS2 | MLXIPL | SIS3 | NR4A1 | SIS4 | FAM83D |
| SIS1 | IL12RB1 | SIS2 | BDH1 | SIS3 | CDA | SIS4 | MYRF |
| SIS1 | CD38 | SIS2 | ACKR2 | SIS3 | SULF1 | SIS4 | ILDR1 |
| SIS1 | PTPRC | SIS2 | ACOX2 | SIS3 | PIGR | SIS4 | BLMH |
| SIS1 | TBC1D10C | SIS2 | HAGH | SIS3 | CLEC4G | SIS4 | SKA1 |
| SIS1 | IKZF3 | SIS2 | KLF15 | SIS3 | TRIM22 | SIS4 | HSP90AB1 |
| SIS1 | BIN2 | SIS2 | SLC46A3 | SIS3 | FGL1 | SIS4 | POGK |
| SIS1 | FCGR2A | SIS2 | GCDH | SIS3 | FBP1 | SIS4 | KRTCAP3 |
| SIS1 | SELL | SIS2 | SULT4A1 | SIS3 | KRT7 | SIS4 | SRC |
| SIS1 | NQO1 | SIS2 | CA14 | SIS3 | PTH1R | SIS4 | ATP1B1 |
| SIS1 | EVI2A | SIS2 | CBR1 | SIS3 | SLC27A2 | SIS4 | IQGAP3 |
| SIS1 | LILRB2 | SIS2 | GSTM1 | SIS3 | MEGF6 | SIS4 | ONECUT1 |
| SIS1 | TRPV2 | SIS2 | PROL1 | SIS3 | SAA4 | SIS4 | HELLS |
| SIS1 | CD4 | SIS2 | PLG | SIS3 | TMEM47 | SIS4 | KIAA1524 |
| SIS1 | CDT1 | SIS2 | SLC2A2 | SIS3 | FHL1 | SIS4 | VTCN1 |
| SIS1 | LCP2 | SIS2 | VSTM4 | SIS3 | CPED1 | SIS4 | WASF1 |
| SIS1 | RASAL3 | SIS2 | PKLR | SIS3 | ANGPTL2 | SIS4 | LBR |
| SIS1 | ITGAX | SIS2 | FABP4 | SIS3 | MMRN2 | SIS4 | HNRNPA1 |
| SIS1 | ADGRE5 | SIS2 | INSIG1 | SIS3 | SPRY1 | SIS4 | C6orf132 |
| SIS1 | FABP3 | SIS2 | THBS4 | SIS3 | MARCO | SIS4 | MEST |
| SIS1 | MYO1F | SIS2 | IGFBP1 | SIS3 | PODXL | SIS4 | TUBB |
| SIS1 | SYK | SIS2 | HPX | SIS3 | ARRDC4 | SIS4 | TGFA |
| SIS1 | HLA-F | SIS2 | F13B | SIS3 | RND3 | SIS4 | MMP7 |
| SIS1 | ZAP70 | SIS2 | IGFBP2 | SIS3 | JUNB | SIS4 | ORC1 |
| SIS1 | ABI3 | SIS2 | ALDOB | SIS3 | CYP8B1 | SIS4 | STMN1 |
| SIS1 | CASP1 | SIS2 | ADRB2 | SIS3 | C8A | SIS4 | SLC6A11 |
| SIS1 | CA9 | SIS2 | FGF21 | SIS3 | TIE1 | SIS4 | CHML |
| SIS1 | CDKN3 | SIS2 | UBE2QL1 | SIS3 | AKR1D1 | SIS4 | IGSF1 |
| SIS1 | MSC | SIS2 | NUDT8 | SIS3 | MGLL | SIS4 | LYPD1 |
| SIS1 | LGALS3BP | SIS2 | ITIH1 | SIS3 | EHD2 | SIS4 | SNRPB |
| SIS1 | KLRB1 | SIS2 | MYCL | SIS3 | RGS5 | SIS4 | MEX3A |
| SIS1 | RUNX3 | SIS2 | COL7A1 | SIS3 | FCN2 | SIS4 | KIAA0101 |
| SIS1 | PLBD1 | SIS2 | ALDH2 | SIS3 | CFHR5 | SIS4 | RPS18 |
| SIS1 | CD44 | SIS2 | AHSG | SIS3 | SYNPO2 | SIS4 | CDC45 |
| SIS1 | CCR1 | SIS2 | CYP3A43 | SIS3 | LBH | SIS4 | SKP2 |
| SIS1 | AKR1B1 | SIS2 | PROC | SIS3 | FXYD6 | SIS4 | RPLP0 |
| SIS1 | SOCS1 | SIS2 | C4BPA | SIS3 | CLEC14A | SIS4 | CDCA2 |
| SIS1 | GSTP1 | SIS2 | SHF | SIS3 | COL12A1 | SIS4 | RMI2 |
| SIS1 | CD247 | SIS2 | MROH2A | SIS3 | HP | SIS4 | MFAP2 |
| SIS1 | CXCL17 | SIS2 | LRCOL1 | SIS3 | KCNE4 | SIS4 | CDH1 |
| SIS1 | HLA-A | SIS2 | AGMO | SIS3 | GSTP1 | SIS4 | ROBO1 |
| SIS1 | IRF1 | SIS2 | TMEM150C | SIS3 | WISP2 | SIS4 | RIBC2 |
| SIS1 | FCMR | SIS2 | TTPA | SIS3 | EFEMP2 | SIS4 | CAPN6 |
